# Neutrophil Extracellular Traps and Platelet Activation for Identifying Severe Episodes and Clinical Trajectories in COVID-19

**DOI:** 10.3390/ijms24076690

**Published:** 2023-04-03

**Authors:** Paula González-Jiménez, Raúl Méndez, Ana Latorre, Mónica Piqueras, María Nieves Balaguer-Cartagena, Antonio Moscardó, Ricardo Alonso, David Hervás, Soledad Reyes, Rosario Menéndez

**Affiliations:** 1 Pneumology Department, La Fe University and Polytechnic Hospital, 46026 Valencia, Spain; 2 Respiratory Infections, Health Research Institute La Fe, 46026 Valencia, Spain; 3 Medicine Department, University of Valencia, 46010 Valencia, Spain; 4 Laboratory Department, La Fe University and Polytechnic Hospital, 46026 Valencia, Spain; 5 Platelet Function Unit, Health Research Institute La Fe, 46026 Valencia, Spain; 6 Data Science, Biostatistics & Bioinformatics, Health Research Institute La Fe, 46026 Valencia, Spain; 7 Department of Applied Statistics and Operational Research and Quality, Universitat Politècnica de València, 46022 Valencia, Spain; 8 Center for Biomedical Research Network in Respiratory Diseases (CIBERES), 28029 Madrid, Spain

**Keywords:** COVID-19, SARS-CoV-2, platelet activation, neutrophil extracellular traps

## Abstract

The role of NETs and platelet activation in COVID-19 is scarcely known. We aimed to evaluate the role of NETs (citrullinated histone H3 [CitH3], cell-free DNA [cfDNA]) and platelet activation markers (soluble CD40 ligand [CD40L] and P-selectin) in estimating the hazard of different clinical trajectories in patients with COVID-19. We performed a prospective study of 204 patients, categorized as outpatient, hospitalized and ICU-admitted. A multistate model was designed to estimate probabilities of clinical transitions across varying states, such as emergency department (ED) visit, discharge (outpatient), ward admission, ICU admission and death. Levels of cfDNA, CitH3 and P-selectin were associated with the severity of presentation and analytical parameters. The model showed an increased risk of higher levels of CitH3 and P-selectin for ED-to-ICU transitions (Hazard Ratio [HR]: 1.35 and 1.31, respectively), as well as an elevated risk of higher levels of P-selectin for ward-to-death transitions (HR: 1.09). Elevated levels of CitH3 (HR: 0.90), cfDNA (HR: 0.84) and P-selectin (HR: 0.91) decreased the probability of ward-to-discharge transitions. A similar trend existed for elevated levels of P-selectin and ICU-to-ward transitions (HR 0.40); In conclusion, increased NET and P-selectin levels are associated with more severe episodes and can prove useful in estimating different clinical trajectories.

## 1. Introduction

The emergence of severe acute respiratory syndrome coronavirus 2 (SARS-CoV-2) led to a worldwide pandemic, named coronavirus disease 2019 (COVID-19) [1]. The clinical presentation of COVID-19 varies in illness severity: asymptomatic or mild in approximately 80% of cases; moderate in 15% and critical in approximately 5%. Factors that explain the differences in illness severity are viral load, vaccination status and, more especially, immune-inflammatory host response [2]. A better knowledge of the pathophysiology and identification of potential therapeutic targets are essential to the development of effective treatments.

Neutrophil extracellular traps (NETs) constitute an innate immune response to snare microorganisms by means of webs, mainly formed by DNA and proteases [3]. NET formation appears because of neutrophil activation triggered by various stimuli, such as microorganisms [4,5], activated platelets [6], damaged endothelium [7] and more. NETs are necessary for an optimal immune response; however, in excess, NETs can lead to lung damage, endothelial injury and microthrombosis, conferring deleterious effects [8]. In previous studies, NET formation was reported in both sera obtained from patients with COVID-19 and pulmonary microvascular lumens gathered during autopsies [9,10].

The interplay between NETs and platelets in infections has been recently recognized. Platelets can identify microorganisms and stimulate neutrophils to produce NETs [6,11]. Conversely, inflammation, damaged endothelium and NETs lead to platelet activation, creating a feedback loop that results in organ damage and microthrombi [12,13]. To date, some small studies have provided valuable information about NETs and platelet activation in COVID-19 [14,15,16,17,18]. Both are crucial to an adequate innate response [13].

We hypothesized that excessive formation of NETs and platelet activation occurs in the most severe episodes of COVID-19, possibly representing early biomarkers for clinical evolution. Determining such aspects may help to estimate the hazard of different clinical scenarios. We aimed to assess initial NETs markers and platelet activation of patients with COVID-19 in the emergency department (ED) and explore their association with varying clinical trajectories.

## 2. Results

### 2.1. Patient Characteristics

The study included 204 patients: 24 outpatients; 172 conventional ward-admitted and eight ICU-admitted within the first 24 h. Table 1 shows the baseline characteristics of the cohort. Patients with initial ICU admission were mainly male, with more underlying cardiovascular risk factors, higher severity (PSI and SOFA scores) and inflammatory response. We observed a high mortality rate in the ward with patients who did not require ICU admission. This may be due to a shortage of ICU beds during the peak of the first wave in Spain and a more restrictive selection regarding ICU admission and patients with many advanced comorbidities and reduced life expectancy [19].

### 2.2. NETs, Platelet Activation and Initial Severity

**Figure 1:**
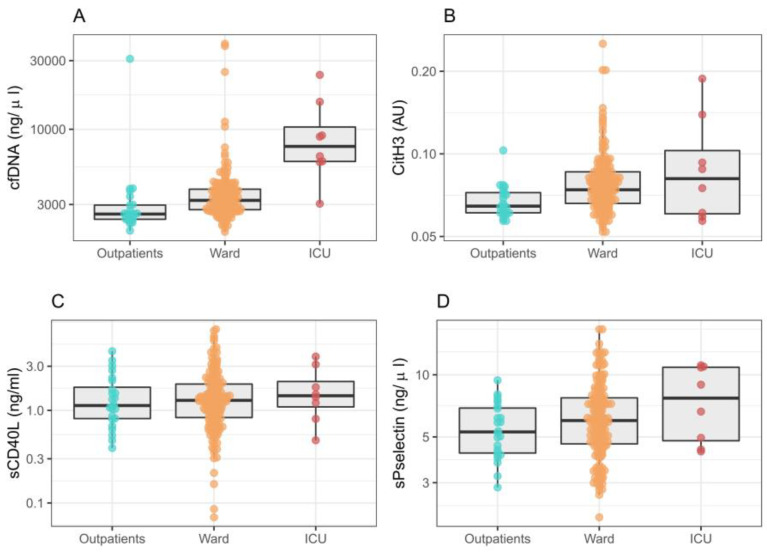
Neutrophil extracellular traps and platelet activation in relation to medical care needed in patients with COVID-19: outpatient, conventional ward admission and initial ICU admission. *p*-value from ordinal regression for discrimination among groups. (**A**) cfDNA levels; OR 6.76 [95% CI 2.95, 17.08], *p* < 0.001. (**B**) CitH3 levels; OR 12.90 [2.69, 62.68], *p* = 0.001. (**C**) sCD40L levels; OR 1.106 [0.654, 1.853], *p* = 0.706. (**D**) sP-selectin levels; OR 2.45 [0.95–6.44], *p* = 0.066. AU: arbitrary units.

CitH3, cfDNA and P-selectin levels were higher in ICU-admitted patients; however, P-selectin levels did not reach statistical significance (*p* = 0.066; OR 2.45 [95% CI 0.95–6.44]) (Figure 1). We found no differences for CD40L (*p* = 0.706; OR 1.106 [0.654, 1.853]). Furthermore, cfDNA showed the best capacity for discrimination among outpatients, ward and ICU admission (*p* < 0.001; OR 6.76 [2.95, 17.08]), followed by CitH3 (*p* = 0.001; OR 12.90 [2.69, 62.68]).

**Figure 2:**
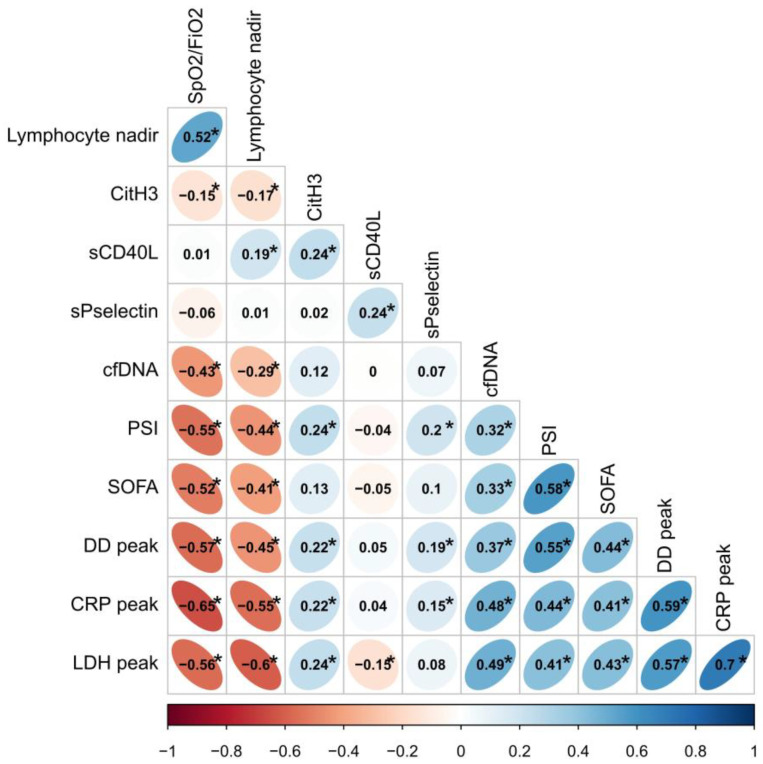
Correlation plot. Neutrophil extracellular traps and platelet activation markers are related to initial severity and prognostic analytical parameters in patients with COVID-19. CRP: C-reactive protein; DD: D-dimer; PSI: pneumonia severity index; SpO_2_/FiO_2_: peripheral blood oxygen saturation/fraction of inspired oxygen; SOFA: sequential organ failure assessment score. We used peak levels of analytical parameters and the lymphocyte count nadir during admission. Correlations were automatically grouped according to similarity. * *p* < 0.05.

Several correlations were calculated between NETs and platelet biomarker levels, and C-reactive protein (CRP) peak during admission, D-dimer (DD) peak, lymphocyte count nadir, SpO2/FiO2, number of lobes with infiltrates and prognostic scales PSI and SOFA scores (Figure 2). Moreover, cfDNA levels were directly correlated with PSI and SOFA scores, yet inversely correlated with SpO2/FiO2. This observation reinforces such association with more severe episodes. CitH3 and P-selectin were also correlated with PSI, yet while inversely correlated with SpO2/FiO2. Additionally, cfDNA and CitH3 levels were found to have a significantly negative correlation with lymphocyte count nadir. With respect to analytical parameters, cfDNA and CitH3 levels were directly correlated with peaks in CRP, LDH and DD. Similar results were found for P-selectin and CRP and DD levels.

We analyzed biomarker levels based on relevant demographics and comorbidities. We found higher levels of NETs in males and the elderly (Table S1), and minor or no differences in biomarker levels based on comorbidities (Table S2).

### 2.3. NETs, Platelet Activation Markers and Clinical Outcomes

**Figure 3:**
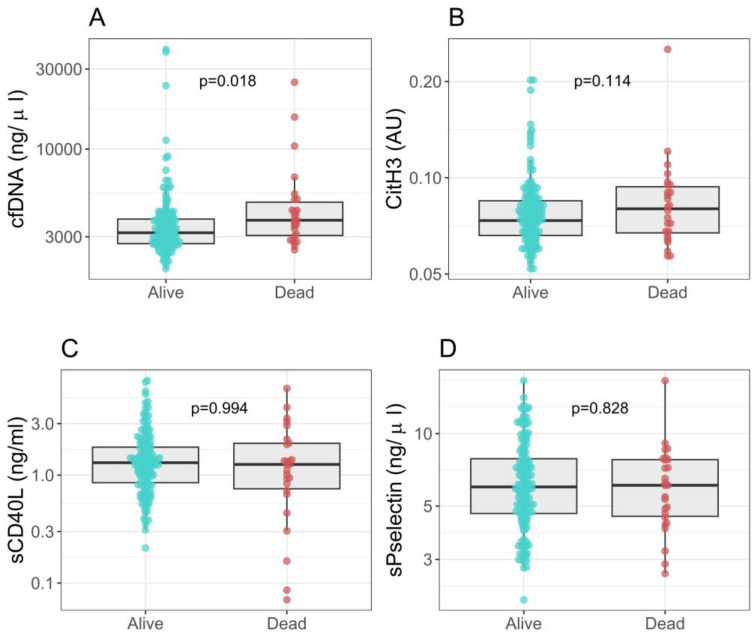
Neutrophil extracellular traps, platelet activation markers and death. (**A**) cfDNA levels; OR 2.27 [1.18, 4.55], *p* = 0.018. (**B**) CitH3 levels; OR 3.19 [0.70, 13.10], *p* = 0.114. (**C**) sCD40L levels; OR 1.00 [0.68, 1.36], *p* = 0.994. (**D**) sP-selectin levels; OR 0.98 [0.83, 1.14], *p* = 0.828. AU: arbitrary units.

During the study, 26 (12.7%) patients died within a median number of 10.5 (5–14) days. Furthermore, 17 of 172 (9.9%) patients initially admitted to the conventional ward were transferred to the ICU. During hospitalization, 15 (7.4%) patients needed invasive mechanical ventilation. In the outpatient group, none required hospital admission or died. Significantly increased levels of cfDNA were found in patients who died (*p* = 0.018; OR 2.27 [1.18, 4.55]) (Figure 3). Table S3 depicts biomarker levels regarding different clinical outcomes, including mortality, the need for supplemental oxygen, respiratory support progression and radiological progression.

We performed several areas under the receiver operating characteristics (AUROC) analyses for biomarkers related to outpatient care, ICU admission (first 24 h) and mechanical ventilation and/or death (Table S4). CitH3 and cfDNA showed the greatest AUROC compared with platelet markers.

### 2.4. NETs and Platelet Activation Markers—A Multistate Model for Allocation and Progression

**Figure 4:**
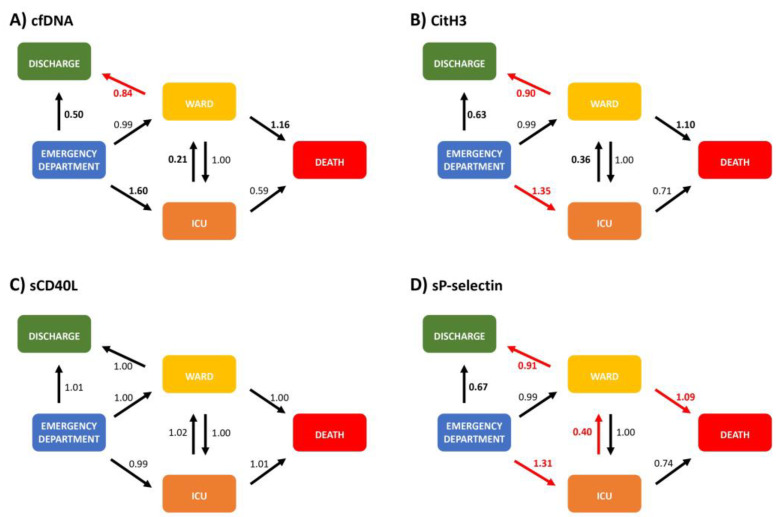
Multistate model for neutrophil extracellular trap and platelet activation markers. Evaluated states were: emergency department (ED) visit, ward admission, intensive care unit (ICU) admission, death and hospital discharge (outpatient). (**A**) cfDNA model. (**B**) CitH3 model. (**C**) sCD40L model. (**D**) sP-selectin model. Arrows show the transitions among states. We show hazard ratios (HR) next to the arrows and represent the increase by one standard deviation above the biomarker level mean. Red arrows and HR indicate statistically significant transitions. HR in bold denotes trends/proximity to statistical significance. Table S2 details 95% confidence intervals. For further explanation, please see text.

Figure 4 shows HRs for transitions among varying clinical states and outcomes according to NETs and platelet biomarker levels. Higher levels of CitH3 and/or P-selectin for ED-to-ICU transitions showed HRs of 1.35 [95% CI 1.05, 4.5] and 1.31 [1.03, 4.69], respectively. Conversely, lower levels of CitH3 (HR 0.90 [0.62, 0.99]) and cfDNA (HR 0.84 [0.45, 0.99]) raised the likelihood of ward-to-discharge transitions. We observed a similar trend for ICU-to-ward transitions (HR 0.36 [0.02, 1.2]; HR 0.21 [0, 1.22]). Furthermore, we reported an increased risk of transitions from ED to ICU (HR 1.31 [1.03, 4.69]) and ward to death (HR 1.09 [1.0, 1.65]) with higher P-selectin levels. Conversely, lower P-selectin levels showed a higher likelihood of transitions from ICU to ward (HR 0.40, 0.0–0.99) and ward to discharge (HR 0.91 [0.75, 0.98]). Table S5 details all HRs and their respective 95% CI.

## 3. Discussion

The main findings of this study were: (i) higher initial levels of cfDNA and CitH3 were associated with more severe clinical presentations that included more elevated scores (SOFA and PSI), hypoxemia and ICU admission. We found a trend for elevated P-selectin levels in patients admitted to the ICU; (ii) cfDNA and CitH3 NETs correlated with lymphopenia and analytical parameters (CRP, LDH and DD) related to COVID-19 progression; (iii) A multistep model was developed to estimate the different clinical trajectories of patients who visited the ED with COVID-19 and had CitH3, cfDNA and P-selectin measured. The model also included an estimation of outcomes related to discharge, ward admission, ICU requirement and death.

The present study comprises patients with varying degrees of COVID-19 severity, from mild-to-moderate to severe. Higher initial systemic NETs levels were found in the most severe patients; that is, those who required ICU admission and presented higher SOFA or PSI scores, lower oxygen saturation and more lobes of pulmonary infiltrates. The involvement of NETs and platelet activation in COVID-19 has been reported in a small group of critically ill patients and autopsies; however, information concerning mild-moderate COVID-19 infection is scarce [20,21,22]. To the authors’ knowledge, there is only one small study (*n* = 36) that has evaluated NET markers and platelet activation simultaneously in patients with COVID-19 [23]. Finally, NETs were also related to the most severe episodes of acute respiratory disease syndrome (ARDS) and community-acquired pneumonia [24].

Levels of CitH3 and cfDNA showed a positive correlation with CRP and DD, indicative of a systemic inflammatory scenario, while a negative correlation was present with lymphocyte count. A common pattern of the neutrophil-to-lymphocyte ratio in community-acquired pneumonia (CAP) and COVID-19 has been previously reported, evaluating the interplay between neutrophils and lymphocytes [25,26,27]. Those analytical findings have been reported in ICU-admitted patients with COVID-19, including in those without prior known comorbidities [28]. Our study builds upon those reports by providing an additional and significant perspective, which is that higher systemic levels of NETs and platelet activation are present in patients with more severe episodes even outside of the ICU. A specific inflammatory response and an immuno-metabolic profile of neutrophils have been found in COVID-19, demonstrating an interplay between inflammation and thrombosis [29]. Increased NETs activity triggers organ damage and endothelial inflammation, drives microthrombosis and provides iterative feedback for inflammation and platelet activation [30]. The relationship between NETs and platelet activation with this prothrombotic state has been demonstrated in several studies, highlighting the involvement of both mechanisms [31].

The clinical course of COVID-19 infection shifts towards progression or resolution based on patient characteristics, age and immunoinflammatory response—even when the initial trigger remains unclear [32,33]. To our knowledge, no previous studies have assessed clinical trajectories of COVID-19 with NETosis and platelet activation. The multistep approach allowed us to estimate transitions among different states and outcomes, that is, from the most favorable, being discharge, to the poorest, being death. This model provides a dynamic approach for interpreting NET production (CitH3 and cfDNA) and P-selectin levels for five clinical states. Higher CitH3 levels were associated with transitions to more severe states, i.e., from ED to ICU, while lower CitH3 levels were associated with more favorable clinical states, i.e., from ward to discharge. Secondly, higher cfDNA (a non-specific marker of NETosis) levels also showed a trend for identifying transitions to more severe states, whereas lower levels were associated with transitions to more favorable clinical states. Finally, higher P-selectin levels were related to more severe clinical trajectories, such as from ED to ICU or ward to death. Conversely, lower P-selectin levels were related to favorable clinical trajectories, such as from ICU to ward or ward to discharge. In the present study, we showed that higher levels of cfDNA, CitH3 and P-selectin, as the expression of increased NETs production and platelet activation, were associated with more unfavorable clinical trajectories. Lower levels were related to more favorable clinical trajectories. Noteworthy, the model was obtained with data acquired at first evaluation, which is more important when identifying whether a subsequent clinical course will shift towards resolution or progression, and understanding when therapeutic decisions could modify outcomes.

Our findings support how the presence of excessive or uncontrolled NETs and platelet activation in COVID-19 can trigger a physiological mechanism called immunothrombosis [34,35]. Immunothrombosis may contribute to increased systemic inflammation [36], which in turn favors platelet activation and NETosis [37,38]. Its inactivation or blockade has been proposed as a possible therapeutic alternative in pneumonia and COVID-19 to break this vicious circle. Encouraging results were indeed shown in preclinical studies exploring platelet response treatments for influenza pneumonia [39]. Promising advances have, thus far, been made in antiplatelet therapy. Many other therapeutic approaches are under development [40]. A recent clinical trial has shown no benefit conferred by combining rivaroxaban with aspirin to treat patients hospitalized for COVID-19. However, this study did not measure NET production or platelet activation. There is a need for new trials to consider these variables in their patient inclusion criteria.

### Potential Limitations

Several limitations must be acknowledged. This is a single-center study, and our findings should be further confirmed. Despite that, this is the largest study evaluating NETs and platelet activation in COVID-19. Second, longitudinal measurements of biomarkers and larger population samples are needed. These findings should, therefore, be considered as hypothesis-generating for the clinical decision-making process. Irrespective, the first evaluation is possibly the most critical phase in the clinical decision-making process. Third, cfDNA is not a specific marker of NETs and could also come from cell death in different types of tissue. Fourth, only soluble platelet activation biomarkers were analyzed and an in vivo analysis at the membrane surface is needed for further elucidation. Lastly, soluble CD40L showed no value. This may be due to the fact that CD40L has multiple functions and is not only specific for platelet activation [41]. In addition, the biological pathways by which the release of one or another platelet marker occurs have not been fully explored. It is plausible that the infection specifically activates only some biological pathways. Moreover, the detection of NETs in human plasma could be achieved through imaging-Flow-Cytometry performed in blood as described by Vats R et al. [42].

## 4. Materials and Methods

### 4.1. Design, Participants and Outcomes

We conducted a prospective, observational study in a tertiary care hospital in Valencia. The Biomedical Research Ethics Committee Hospital La Fe approved the study (2020-282-1). The informed consent was waived, as the remaining samples from the regular laboratory were used. We recruited patients who visited the emergency department (ED) at some point between 20 March and 27 April with a confirmed case of COVID-19 using reverse transcription polymerase chain reaction (RT-PCR) testing of nasopharyngeal swabs or sputum samples. Patients were divided into three groups: (a) patients treated on an outpatient basis; (b) patients admitted to the conventional ward and (c) patients admitted to the intensive care unit (ICU) within the first 24 h. Relevant demographics, comorbidities, chronic treatments, analytical parameters and microbiological and radiographic data were recorded. Initial severity was measured with the Pneumonia Severity Index (PSI) and Sequential Organ Failure Assessment (SOFA) score.

Evaluated outcomes comprised different clinical states, including ED, conventional ward or ICU admission, in-hospital mortality or discharge as an outpatient. All hospitalized patients were monitored until discharge or death, or in the case of outpatients, for up to 30 days.

### 4.2. Blood Samples

Peripheral venous blood was drawn from patients during ED visits and stored in ethylenediaminetetraacetic acid (EDTA) tubes. EDTA tubes were centrifugated (1258× *g*) for 10 min to obtain plasma and aliquoted for storage at −80 °C until analysis.

### 4.3. Neutrophil Extracellular Traps

Detection of citrullinated histone H3 (CitH3) and cell-free DNA (cfDNA) was performed as previously described [43]. CitH3 is the most specific marker of NET formation [44]. To capture histones, plasma samples were mixed with a monoclonal mouse anti-histone biotinylated antibody of Cell Death Detection ELISA^PLUS^ (Inmunoreagent 7600 µL of Incubation Buffer + 400µL of Anti-histone-biotin) in a streptavidin-coated microplate. After the solution was removed, wells were washed 3 x with 300 µL of Incubation Buffer. Secondly, a rabbit polyclonal anti-histone-H3 antibody (1 mg/mL) (citrullinated R17 + R2 + R8) (ab81797; Abcam Inc., Waltham, MA, USA) was diluted 1:1000 with Incubation Buffer and incubated overnight at 4 °C with constant stirring. After rinsing 3 x with 300 µL of Incubation Buffer, the detection was performed with a peroxidase-linked antibody (n° ref NA-9340, GE Biosciences, Barcelona, Spain) diluted 1:500 with Incubation Buffer and incubated for 1 h at 25 °C. Wells were carefully washed with Incubation Buffer. For development, the microplate was incubated with ABTS Solution for approximately 20 min. The reaction was stopped with the addition of ABTS Stop solution; it was measured at 405 nm. Values were normalized to a pool of samples from normal subjects, included in all microplates, and expressed as individual absorption values [43].

To determine cfDNA, a marker of cell death and a non-specific component of NETs, plasma was diluted 1:10 with phosphate-buffered saline (PBS [in mmol/L: NaCl 137, KCl 2.7, Na_2_HPO_4_ 10, KH_2_PO_4_, pH 7.4]) and mixed with an equal volume of 1 mM SytoxGreen (n° ref: S7020, Invitrogen, Carlsbad, CA, USA). Fluorescence was determined in a fluorescence microplate reader (Ex = 485 nm; Em = 538 nm) (Gemini XPS; Molecular Devices, Sunnyvale, CA, USA). A calibration curve was generated with calf thymus DNA [10 mg/mL] (n° ref: 15633-019, Invitrogen) in PBS.

### 4.4. Platelet Activation

Soluble CD40L and P-selectin were measured as recognized platelet activation markers related to endothelial damage, inflammation and neutrophil activation [7,45]. Plasma CD40L levels were measured with the Human CD40L Instant ELISA (Affymetrix eBioscience). Plasma samples were diluted 1:5 with distilled water. Absorbance readings were obtained with a spectrophotometer using 450 nm; concentration (ng/mL) was calculated with a standard curve for Human sCD40L Instant ELISA.

To assess platelet activation, plasma P-selectin levels were also measured with commercially available enzyme-linked immunosorbent assay (ELISA) kits from Affymetrix (eBioscience) [46]. Plasma was diluted 1:10 with a sample diluent. Absorbance readings were obtained with a spectrophotometer using 450 nm; concentration (ng/mL) was calculated with a standard curve for Human P-selectin ELISA.

### 4.5. Statistical Analysis

Data were summarized as N (%) or median (1st quartile, 3rd quartile) when appropriate. Evaluated states were ED visit, ward admission, ICU admission, death and hospital discharge. Prior to modelling, biomarker values were log-transformed. Ordinal regression models were used to assess associations between patient groups and each biomarker. Correlations between analytical parameters and studied biomarkers were estimated using Spearman’s rank correlation. The multistate model, including ED, outpatient, ward, ICU and death as states and biomarker values as predictors were adjusted for prognostic evaluation (Figure S1). Predictor variables were scaled to unit variance and centered at zero to adjust the multistate model. Results were summarized as a hazard ratio [95% confidence interval]. Confidence intervals were estimated using bias-corrected, accelerated bootstrap intervals with 200 bootstrap replicates. The HR represents an increase by one standard deviation above the biomarker level mean. Statistical significance was considered if *p* < 0.05. Statistical analyses were performed using R (version 4.0.2) and R packages clickR (0.4.47), ordinal (2019.12-10) and mstate (0.2.12).

## 5. Conclusions

Our findings provided relevant information about NET production and platelet activation in COVID-19. Increased levels of CitH3 and/or cfDNA—as an expression of NET production—and P-selectin are related to initial severity and poor outcomes. NETs were associated with death and other important outcomes, such as discharge or disease progression, implying their crucial role in determining subsequent clinical trajectories. NET expression and P-selectin systemic determinations could serve as an additional tool in personalizing initial assessments of COVID-19 severity and designing trials for targeted therapeutic regimes aimed at modulating excessive production capable of triggering endothelial and organ damage.

## Figures and Tables

**Table 1:** Patient Characteristics Related to Baseline, Severity, Treatment Received, Analytical Parameters, Level of Respiratory Support and Outcomes.

Characteristic	All (*n* = 204)	Outpatients (*n* = 24)	Ward (*n* = 172)	ICU (First 24 h) (*n* = 8)
Age, years, median (1st quartile, 3rd quartile)	64 (51, 77)	49 (39, 58)	66 (53, 79)	63 (54, 66)
			
Distribution, no. (%)				
<50 years	5 (22.1)	12 (50)	32 (18.6)	1 (12.5)
50 to <70 years	86 (42.2)	10 (41.7)	69 (40.1)	7 (87.5)
≥70 years	73 (35.8)	2 (8.3)	71 (41.3)	0 (0)
Male sex, no. (%)	109 (53.4)	4 (16.7)	98 (57)	7 (87.5)
Co-existing conditions, no. (%)				
Any	136 (66.7)	15 (62.5)	116 (67.4)	5 (62.5)
Hypertension	86 (42.2)	7 (29.2)	74 (43)	5 (62.5)
Diabetes	46 (22.5)	1 (4.2)	42 (24.4)	3 (37.5)
Dyslipidemia	62 (30.4)	4 (16.7)	55 (32)	3 (37.5)
Chronic heart disease	25 (12.3)	2 (8.3)	23 (13.4)	0 (0)
Chronic renal disease *	27 (13.2)	0 (0)	27 (15.7)	0 (0)
Chronic liver disease	6 (2.9)	0 (0)	6 (3.5)	0 (0)
Neurological disease	25 (12.3)	1 (4.2)	24 (14)	0 (0)
Chronic respiratory disease	23 (11.3)	5 (20.8)	18 (10.5)	0 (0)
No. of days since symptom onset, median (1st quartile, 3rd quartile) ^†^	7 (5, 10)	10 (5, 15)	7 (5, 10)	5 (3, 6)
SpO_2_/FiO_2_ at admission (1st quartile, 3rd quartile)	452.4 (438.1, 461.9)	466.7 (461.9, 471.4)	452.4 (438.1, 457.1)	264.3 (168, 326)
Radiological data at admission				
Bilateral infiltrates, no. (%)	112 (63.6)	0 (0)	104 (65)	8 (100)
Severity				
PSI score, median (1st quartile, 3rd quartile)	68 (48, 91)	42 (32, 53)	71 (51, 95)	89 (75, 101)
I-III, no. (%)	151 (74.4)	24 (100)	123 (71.9)	4 (50)
IV-V, no. (%)	52 (25.6)	0 (0)	48 (28.1)	4 (50)
SOFA score, median (1st quartile, 3rd quartile)	1 (0, 2)	0 (0, 0)	1 (0, 2)	2 (1, 4)
Analytical parameters				
Peak LDH, UI/L, median (1st quartile, 3rd quartile)	295.5 (238, 420)	170 (156, 225.5)	310 (249, 423)	494 (427.5, 674.5)
Peak C-reactive protein, mg/L, median (1st quartile, 3rd quartile)	87.2 (31.3, 200.9)	5.7 (1.4, 12.2)	104.7 (41.6, 205.1)	372.2 (302.9, 530.3)
Lymphocyte count nadir, cells/mL, median (1st quartile, 3rd quartile)	910 (640, 1305)	1500 (1075, 2175)	860 (640, 1245)	480 (425, 610)
Peak D-dimer, ng/mL, median (1st quartile, 3rd quartile) **	958.5 (477, 2143)	353 (215, 567)	1014 (527, 2143)	25,677 (11,486, 43,961)
Respiratory support, no. (%)				
O2 nasal cannula, no. (%)	19 (8.1)	0 (0)	19 (11)	0 (0)
O2 Venturi or reservoir mask, no. (%)	55 (23.5)	0 (0)	55 (32)	0 (0)
HFNC/CPAP, no. (%)	10 (4.3)	0 (0)	10 (5.8)	0 (0)
MV, no. (%)	15 (6.4)	0 (0)	7 (4.1)	8 (100)
Median length of MV, days, median (1st quartile, 3rd quartile)	NA	NA	12 (11, 14)	13.5 (11, 16.5)
Outcomes and complications				
Median length of hospital stay, days, median (1st quartile, 3rd quartile)	12 (9, 20.5)	NA	12 (9, 19)	32.5 (18, 45)
Transfer to ICU from ward, no. (%) ^††^	17 (8.3)	NA	17 (9.9)	NA
In-hospital mortality, no. (%)	26 (12.7)	0 (0)	24 (14)	2 (25)

ACE denotes angiotensin-converting enzyme; HFNC/CPAP: high-flow nasal cannula/continuous positive airway pressure; ICU: intensive care unit; LDH: lactate dehydrogenase; MV: mechanical ventilation; NA: not applicable; NIV: non-invasive ventilation; SpO2/FiO2: peripheral blood oxygen saturation/fraction of inspired oxygen. * Stage ≥ 2. ^†^ Missing data in 8 patients. ** Missing data in two patients. ^††^ Need for ICU admission > 24 h after hospitalization.

## Data Availability

The datasets used and/or analyzed during the current study are available from the corresponding author on reasonable request.

## Supplementary Materials

The following supporting information can be downloaded at: https://www.mdpi.com/article/10.3390/ijms24076690/s1, Table S1. Biomarker levels and demographics; Table S2. Biomarker levels and comorbidities; Table S3. Biomarker levels and clinical outcomes; Table S4. Area Under the Receiver Operating Characteristics Analysis; Table S5. Multistate model; Figure S1. Multistate model.
