# Supplementary material for: Neutrophil Extracellular Traps and Platelet Activation for Identifying Severe Episodes and Clinical Trajectories in COVID-19
Source: Int J Mol Sci. 2023 Apr 3;24(7):6690. doi: 10.3390/ijms24076690 (PMC10094814; doi:10.3390/ijms24076690)
Supplement: Supplementary file 1 [file ijms-24-06690-s001.zip › supplemental-file.pdf]

**SUPPLEMENTAL FILE**

# Neutrophil Extracellular Traps and Platelet Activation for Identifying Severe Episodes and Clinical Trajectories in COVID-19

Paula González-Jiménez[1,2,3,*], Raúl Méndez[1,2,*], Ana Latorre[2], Mónica Piqueras[3,4], Mari Nieves Balaguer-Cartagena[1], Antonio Moscardó[5], Ricardo Alonso[4], David Hervás[6,7], Soledad Reyes[1,2], Rosario Menéndez[1,2,3,8]

[1]Pneumology Department. La Fe University and Polytechnic Hospital. Valencia, Spain.

[2]Respiratory Infections. Health Research Institute La Fe. Valencia, Spain.

[3]University of Valencia. Valencia, Spain.

[4]Laboratory Department. La Fe University and Polytechnic Hospital. Valencia, Spain.

[5]Platelet Function Unit. Health Research Institute La Fe. Valencia, Spain.

[6]Data Science, Biostatistics & Bioinformatics. Health Research Institute La Fe. Valencia, Spain.

[7]Department of Applied Statistics and Operational Research and Quality. Universitat Politècnica de València. Valencia, Spain.

[8]Center for Biomedical Research Network in Respiratory Diseases (CIBERES). Madrid, Spain.

*Equal contribution

**Corresponding author:** Raúl Méndez

Servicio de Neumología. Hospital Universitario y Politécnico La Fe,

Avda. Fernando Abril Martorell 106, 46026 Valencia. Spain,

e-mail: mendez_rau@gva.es

Table S1. Biomarker levels and demographics.

| | Age ≤ 65 years (N=114) | Age > 65 years (N=90) | P |
|---|---|---|---|
| CitH3 (AU) | 0.071 [0.064, 0.082] | 0.077 [0.067, 0.089] | 0.027 |
| cfDNA (ng/uL) | 2997 [2574, 3840] | 3334 [2802, 3887] | 0.122 |
| sCD40L (ng/mL) | 1.33 [0.84, 1.78] | 1.25 [0.81, 1.94] | 0.877 |
| sP-selectin (ng/mL) | 5.76 [4.51, 7.20] | 6.63 [4.49, 8.97] | 0.031 |
| | **Female (N=95)** | **Male (N=109)** | **P** |
| CitH3 (AU) | 0.071 [0.063, 0.079] | 0.076 [0.066, 0.088] | 0.022 |
| cfDNA (ng/uL) | 2958 [2571, 3739] | 3362 [2841, 4084] | 0.004 |
| sCD40L (ng/mL) | 1.26 [0.93, 2.04] | 1.30 [0.80, 1.82] | 0.627 |
| sP-selectin (ng/mL) | 5.89 [4.49, 7.29] | 5.92 [4.51, 7.89] | 0.475 |

Data is summarised as median [$1^{st}$ quartile, $3^{rd}$ quartile].

Table S2. Biomarker levels and comorbidities.

| | No Chronic Heart Disease (N=179) | Chronic Heart Disease (N=25) | P |
|---|---|---|---|
| CitH3 (AU) | 0.073 [0.064, 0.085] | 0.073 [0.066, 0.079] | 0.769 |
| cfDNA (ng/uL) | 3152 [2689, 3840] | 3143 [2781, 4055] | 0.739 |
| sCD40L (ng/mL) | 1.30 [0.84, 1.93] | 1.11 [0.68, 1.93] | 0.469 |
| sP-selectin (ng/mL) | 5.91 [4.49, 7.63] | 6.26 [4.72, 8.61] | 0.439 |
| | **No Diabetes (N=158)** | **Diabetes (N=46)** | **P** |
| CitH3 (AU) | 0.073 [0.064, 0.085] | 0.074 [0.065, 0.082] | 0.506 |
| cfDNA (ng/uL) | 3053 [2669, 3754] | 3505 [2805, 4075] | 0.147 |
| sCD40L (ng/mL) | 1.24 [0.80, 1.68] | 1.49 [0.99, 2.21] | 0.016 |
| sP-selectin (ng/mL) | 5.89 [4.49, 7.54] | 6.39 [4.51, 7.89] | 0.332 |
| | **No Hypertension (N=118)** | **Hypertension (N=86)** | **P** |
| CitH3 (AU) | 0.071 [0.064, 0.081] | 0.077 [0.066, 0.089] | 0.045 |
| cfDNA (ng/uL) | 2981 [2654, 3840] | 3328 [2802, 3948] | 0.177 |
| sCD40L (ng/mL) | 1.29 [0.84, 1.77] | 1.27 [0.81, 1.98] | 0.845 |
| sP-selectin (ng/mL) | 5.79 [4.46, 7.54] | 6.23 [4.73, 8.47] | 0.137 |
| | **No Dyslipidemia (N=142)** | **Dyslipidemia (N=62)** | **P** |
| CitH3 (AU) | 0.073 [0.064, 0.084] | 0.073 [0.065, 0.086] | 0.769 |
| cfDNA (ng/uL) | 3056 [2656, 3829] | 3350 [2781, 4045] | 0.191 |
| sCD40L (ng/mL) | 1.26 [0.87, 1.93] | 1.32 [0.80, 1.82] | 0.713 |
| sP-selectin (ng/mL) | 5.91 [4.49, 7.56] | 6.03 [4.65, 8.71] | 0.431 |

Data is summarised as median [1st quartile, 3rd quartile].

Table S3. Biomarker levels and clinical outcomes

| | No Mortality (N=178) | Mortality (N=26) | P |
|---|---|---|---|
| CitH3 (AU) | 0.073 [0.064, 0.082] | 0.080 [0.067, 0.095] | 0.131 |
| cfDNA (ng/uL) | 3056 [2656, 3753] | 3764 [3038, 5004] | 0.002 |
| sCD40L (ng/mL) | 1.29 [0.84, 1.82] | 1.23 [0.72, 1.99] | 0.572 |
| sP-selectin (ng/mL) | 5.91 [4.51, 7.63] | 6.1 [4.49, 7.8] | 0.849 |
| | **No supplemental oxygen (N=104)** | **Supplemental oxygen (N=100)** | **P** |
| CitH3 (AU) | 0.071 [0.064, 0.078] | 0.077 [0.067, 0.091] | 0.003 |
| cfDNA (ng/uL) | 2848 [2555, 3451] | 3608 [2968, 4260] | <0.001 |
| sCD40L (ng/mL) | 1.33 [0.81, 2.08] | 1.25 [0.86, 1.75] | 0.575 |
| sP-selectin (ng/mL) | 5.72 [4.49, 7.26] | 6.28 [4.58, 7.91] | 0.388 |
| | **No radiological progression (N=131)** | **Radiological progression (N=73)** | **P** |
| CitH3 (AU) | 0.072 [0.064, 0.082] | 0.076 [0.067, 0.090] | 0.054 |
| cfDNA (ng/uL) | 2976 [2583, 3745] | 3552 [2945, 4172] | <0.001 |
| sCD40L (ng/mL) | 1.32 [0.87, 2.15] | 1.21 [0.72, 1.73] | 0.125 |
| sP-selectin (ng/mL) | 5.87 [4.46, 7.54] | 6.26 [4.67, 7.91] | 0.537 |
| | **No respiratory support progression (N=133)** | **Respiratory support progression (N=71)** | **P** |
| CitH3 (AU) | 0.072 [0.064, 0.081] | 0.075 [0.066, 0.095] | 0.041 |
| cfDNA (ng/uL) | 2933 [2582, 3623] | 3621 [2982, 4324] | <0.001 |
| sCD40L (ng/mL) | 1.34 [0.84, 2.05] | 1.19 [0.81, 1.73] | 0.232 |
| sP-selectin (ng/mL) | 5.72 [4.49, 7.23] | 6.53 [4.67, 8.05] | 0.145 |

Data is summarised as median [$1^{st}$ quartile, $3^{rd}$ quartile].

Table S4. Area Under the Receiver Operating Characteristics Analysis.

| **Biomarker** | **Outpatients** | **ICU (first 24 h)** | **MV and/or death** |
|---|---|---|---|
| CitH3 | 0.296 [0.199, 0.393]** | 0.555 [0.286, 0.823] | 0.608 [0.493, 0.722]* |
| cfDNA | 0.247 [0.131, 0.362]*** | 0.904 [0.787, 1.000]*** | 0.744 [0.657, 0.831]*** |
| sCD40L | 0.479 [0.355, 0.603] | 0.574 [0.372, 0.777] | 0.477 [0.369, 0.584] |
| sP-selectin | 0.408 [0.301, 0.515] | 0.622 [0.407, 0.836] | 0.546 [0.441, 0.651] |

Data presented as AUROC [95% confidence interval]. AUROC denotes Area Under the Receiver Operating Characteristics; ICU: intensive care unit; MV: mechanical ventilation. *p<0.05, **p<0.01, ***p<0.001

Table S5. Multistate model.

| Biomarker | Transition | Hazard Ratio | 95% Confidence Interval |
|---|---|---|---|
| CitH3 | ED -> Discharge | 0.64 | [0.08, 1.06] |
| cfDNA | ED -> Discharge | 0.50 | [0.00, 1.22] |
| sCD40L | ED -> Discharge | 1.01 | [0.71, 1.51] |
| sP-selectin | ED -> Discharge | 0.67 | [0.26, 1.06] |
| CitH3 | ED -> Ward | 0.99 | [0.81, 1.09] |
| cfDNA | ED -> Ward | 0.99 | [0.64, 1.17] |
| sCD40L | ED -> Ward | 1.00 | [0.95, 1.08] |
| sP-selectin | ED -> Ward | 0.99 | [0.78, 1.05] |
| CitH3 | ED -> ICU | 1.35 | [1.05, 4.52] |
| cfDNA | ED -> ICU | 1.59 | [0.91, 2.99] |
| sCD40L | ED -> ICU | 0.99 | [0.44, 1.49] |
| sP-selectin | ED -> ICU | 1.31 | [1.03, 4.69] |
| CitH3 | Ward -> Discharge | 0.90 | [0.62, 0.99] |
| cfDNA | Ward -> Discharge | 0.84 | [0.48, 0.99] |
| sCD40L | Ward -> Discharge | 1.00 | [0.92, 1.17] |
| sP-selectin | Ward -> Discharge | 0.91 | [0.75, 0.99] |
| CitH3 | Ward -> ICU | 1.00 | [0.68, 1.21] |
| cfDNA | Ward -> ICU | 1.00 | [0.76, 1.34] |
| sCD40L | Ward -> ICU | 1.00 | [0.92, 1.11] |
| sP-selectin | Ward -> ICU | 1.00 | [0.79, 1.18] |
| CitH3 | Ward -> Death | 1.10 | [0.99, 1.61] |
| cfDNA | Ward -> Death | 1.16 | [0.94, 2.02] |
| sCD40L | Ward -> Death | 1.00 | [0.84, 1.10] |
| sP-selectin | Ward -> Death | 1.09 | [1.00, 1.65] |
| CitH3 | ICU -> Ward | 0.36 | [0.02, 1.21] |
| cfDNA | ICU -> Ward | 0.20 | [0.00, 1.22] |
| sCD40L | ICU -> Ward | 1.01 | [0.15, 16.10] |
| sP-selectin | ICU -> Ward | 0.40 | [0.00, 0.99] |
| CitH3 | ICU -> Death | 0.72 | [0.00, 1.27] |
| cfDNA | ICU -> Death | 0.59 | [0.00, 1.42] |
| sCD40L | ICU -> Death | 1.00 | [0.53, 13.73] |

| | | | |
|---|---|---|---|
| sP-selectin | ICU -> Death | 0.74 | [0.00, 1.04] |

ED denotes emergency department; ICU: intensive care unit.

## FIGURE LEGENDS SUPPLEMENTAL FILE

**Figure S1.** Multistate model.
